# Impaired Pre-mRNA Processing and Altered Architecture of 3′ Untranslated Regions Contribute to the Development of Human Disorders

**DOI:** 10.3390/ijms140815681

**Published:** 2013-07-26

**Authors:** Eva Michalova, Borivoj Vojtesek, Roman Hrstka

**Affiliations:** Regional Centre for Applied Molecular Oncology, Masaryk Memorial Cancer Institute, Zluty kopec 7, Brno 656 53, Czech Republic; E-Mails: michalova@mou.cz (E.M.); vojtesek@mou.cz (B.V.)

**Keywords:** 3′ UTR, trinucleotide repeat expansion, polymorphism, mutation, cancer

## Abstract

The biological fate of each mRNA and consequently, the protein to be synthesised, is highly dependent on the nature of the 3′ untranslated region. Despite its non-coding character, the 3′ UTR may affect the final mRNA stability, the localisation, the export from the nucleus and the translation efficiency. The conserved regulatory sequences within 3′ UTRs and the specific elements binding to them enable gene expression control at the posttranscriptional level and all these processes reflect the actual state of the cell including proliferation, differentiation, cellular stress or tumourigenesis. Through this article, we briefly outline how the alterations in the establishment and final architecture of 3′ UTRs may contribute to the development of various disorders in humans.

## 1. Introduction

According to the central dogma of molecular biology, proteosynthesis proceeds from the genetic information carried by a DNA sequence through its transcription to RNA that functions as a template for polypeptide synthesis during the follow-up translation step [1]. Therefore, RNA might represent an interface between the coding DNA and the final protein. Recently it has also become known that RNAs hold various functions in the cell and not all the RNAs are synthesised as mRNA templates for future polypeptides.

Transcription transfers the genetic information from a coding gene to a primary transcript—A precursor-mRNA (pre-mRNA). Normally, pre-mRNAs undergo a series of posttranscriptional modifications in the nucleus which bring forth the mRNA. These processing steps include capping of the 5′ end, removal of introns by splicing, endonucleolytical cleavage and polyadenylation of the 3′ end and editing. mRNAs are finally assembled by a central coding region which is translated to the final polypeptide and by non-coding regions at 5′ and 3′ ends that are not translated (untranslated regions; UTRs).

During the last decade, UTRs have been shown to harbour various sequence motifs (*cis*-acting elements, *cis*-elements) that in cooperation with specific binding proteins or RNAs (*trans*-acting elements, *trans*-elements) regulate the proper posttranscriptional modifications and proteosynthesis. Together with the pre-mRNA processing mechanism, these regulations play important roles in maintaining cellular functions reflecting cellular proliferation or differentiation and participating in cellular reactions to stress stimuli. Therefore, any alteration in the processing steps, mutation or polymorphism can influence the final character of the mRNA 3′ end, the functional properties of the 3′ UTR and the fate of the mRNA leading to variety of disorders in humans including cancer.

## 2. Processing of Pre-mRNA 3′ End

### 2.1. Polyadenylation Signal

The cleavage and the addition of a poly(A) tail to the 3′ end of a pre-mRNA are crucial for an efficient transcription termination, mRNA stability and export to the cytoplasm where the polypeptide chain is synthesised on the ribosomes [2,3]. The polyadenylation signal (PAS), a hexamer “AAUAAA” (less frequently—app. 15%—“AUUAAA”), located approximately 10–30 nucleotides (nt) upstream of the cleavage site, was identified as a highly conserved signal for the endonucleolytic cleavage at the 3′ end [4]. Although additional sequences are recognised to stimulate the cleavage reaction [5], the PAS remains crucial for this process. Therefore, alterations in PAS sequence (SNP, insertions/deletions) disrupt the cleavage and polyadenylation steps resulting in various pathologies in humans, including an association with different malignancies. For instance, modified hexamers were found to inactivate gene expression in globin coding genes (“AAUAAG” in α2-globin, “AACAAA” in β-globin) of thalassaemia patients [6,7]. Similarly, a transition “AAUAAA”→“AAUGAA” within the PAS of *Foxp3* (forkhead box P3) transcript contributes to the IPEX syndrome (Immunodysregulation, Polyendocrinopathy, and Enteropathy, X-linked), a fatal autoimmune disease by reduced levels of Foxp3 transcription factor leading to the dysfunction of regulatory T cells [8]. The polymorphism of PAS in the human *N*-acetyltransferase coding gene (*NAT1*) influences the acetylation of carcinogens and administered drugs. Conversely, the T→A transversion modifying the polyadenylation signal (“AAUAAA”→“AAAAAA”) within the NAT1*10 pre-mRNA does not significantly change the final protein level or the catalytic activity as shown previously in bladder and colon tissues [9]. However, the insertion of “AAA” to the 3′ side of the PAS in the mutant allele NAT1*16 results in a significant decrease of the protein level and of the catalytic activity measured *in vitro* suggesting that the cause lies in the disrupted secondary structure of the mRNA [10]. The human serotonin transporter terminates the neurotransmission by the reuptake of serotonin and the alteration in the transporter coding gene (*hSERT*) is potentially involved in the development of the affective disorder [11]. Two different polyadenylation sites “AATGAA” and “AG/TTAAC” were identified within *hSERT*, and this polymorphism was considered to be playing role in this event, however it did not correlate with the susceptibility to the affective disorder [12]. Human lysosomal alpha-galactosidase A, the enzyme responsible for glycosphingolipid catabolism, bears the PAS within the coding sequence of the *GAL* gene and its mRNA thus lacks the 3′ UTR [13]. The deletion of the “AA” dinucleotide within the PAS results in deficient enzymatic activity (residual or null) of the protein and in the development of Fabry disease, an inborn X-linked disorder characterised by the accumulation of globotriaosylceramide (GL-3), particularly, in vascular endothelial cells throughout the body [14]. Fabry disease patients develop vasculopathy and their life expectancy is shortened due to the renal insufficiency, cardiac disease and stroke [15,16].

### 2.2. Cleavage and Polyadenylation

Numerous proteins grouped into functional protein complexes participate in 3′ end processing. The cleavage and polyadenylation specificity factor (CPSF) consists of five subunits: CPSF-160, -100, -73, -30 and hFip1. It recognises the PAS by the CPSF-160 subunit and catalyses the cleavage reaction through the CPSF-73 subunit [2,17]. Cleavage factors I and II recognise the additional sequence elements required for 3′ end processing. They provide an interaction with poly(A) polymerase (PAP) and with nuclear poly(A) binding protein (PABPN1) and stimulate the cleavage reaction [18,19]. PAP catalyses the addition of a poly(A) tail under the control of PABPN1 [20].

The pre-mRNA 3′ end is cleaved at the pA site, preferentially after the “CA” dinucleotide, however variations were observed, e.g., in the prothrombin gene (coagulation factor II, F2) where the cleavage normally occurs after the “CG” dinucleotide [21]. The cleavage reaction after the “CG” dinucleotide was observed to be less productive *in vitro* [22]. When mutation “CG”→“CA” occurs in F2 mRNA, this more effective 3′ end processing leads to an increased concentration of F2 factor in plasma, resulting in a higher risk for thrombosis development [23].

The poly(A) tail comprising approximately 250 A-nucleotides in mammals is attached to the primary transcript at the cleaved pA site by a protein complex with PAP. The poly(A) sequence protects the 3′ end against degrading exonucleases and enables the export of mRNA to the cytoplasm. It is also essential for the transcription termination, and together with the 5′ cap and related binding proteins it enhances the translation process. The emerging poly(A) sequence is recognised and bound by PABPN1, which needs at least 27 A-nucleotides for a stable protection of the 3′ end against nucleases [24]. PABPN1 is an ubiquitously expressed protein which binds to and regulates the processivity of PAP (that normally shows a low affinity for RNA substrates) and thus controls the elongation of poly(A) tail [25]. Normally, 10× “GCN” repeats coding for alanines within the *N*-terminus of the protein are present at the 5′ end of the first exon of *PABPN1* gene. The expansion to 12–17× “GCN” repeats results in the synthesis of a misfolded protein that aggregates as filaments in nuclear inclusions in skeletal muscle fibres leading to cell death [26]. Patients with this expansion of a polyalanine stretch develop oculopharyngeal muscular dystrophy (OPMD), an autosomal dominant muscle disease. OPMD usually occurs at age fifty and manifests by eyelid dropping, mild ophthalmoplegia, dysphagia and generalised muscle weakness and atrophy. Mechanistically, the deficit of functional PABPN1 affects other pre-mRNA processing steps: polyadenylation, mRNA export to cytoplasm and mRNA stability. Recently PABPN1 was identified to be involved in the regulation of alternative cleavage and polyadenylation (APA) [27]. It is becoming evident that a large portion of human genes contain multiple cleavage sites and PASs in their 3′ UTRs generating multiple mRNA isoforms with different 3′ UTRs [28–30]. The choice of an alternative pA site determines the length of the 3′ UTR and furthermore the stability, localisation and translation efficiency of mRNA [31]. On the basis of the results of multiple studies, the length of 3′ UTRs is inversely correlated with mRNA stability, gene expression and cellular proliferation. Mutated PABPN1 strongly correlates with usage of an alternative pA site and therefore deregulated gene expression [32]. An increased level of mutated protein causes cellular stress *in vitro*, enhances the expression of pro-apoptotic proteins and induces apoptosis in a p53 dependent manner [33].

## 3. Repeat Expansion Disorders

Human repeat expansion disorders represent an extensive heterogeneous group of diseases that are caused by the pathological expansion of repeats (mostly trinucleotide) in the coding or non-coding sequences of specific loci. The non-coding repeat expansions cause multisystem diseases and the extent of the repetitions often reflects disease severity and age of onset. The basis of the pathological mechanism of the non-coding repeats expansions thus lies at the posttranscriptional control of the gene expression and the resulting disorders thus mostly show similar aspects. As our review is focused on the role of 3′ UTRs, we describe only selected disorders related to this topic.

### 3.1. Myotonic Dystrophy Type 1

Myotonic dystrophy type 1 (DM1) is an inherited autosomal dominant disease that is caused by the presence of multiple “CUG” repeats within the 3′ UTR of myotonic dystrophy protein kinase (*DMPK*) pre-mRNA [34,35]. The number of “CUG” repeats strongly affects the final manifestation of the disease and the age-at-onset and varies from 5 to 35 repeats representing normal alleles, through 50–150 repeats in unaffected individuals or patients with mild to classical syndromes, up to more than 150 repeats in patients with severe DM1 [36]. When more than 1000 repeats are present, foetal development is disrupted and congenital DM1 occurs [37]. Moreover, a higher number of repeats implies an increased inherited instability of the mutant locus and further increase in copies transmitted across generations [38]

“CUG” transcripts were originally thought to cause DM1 pathogenesis when detected in a form of RNA foci in the nuclei of DM1 cells due to a blocked export of mutated transcripts to the cytoplasm [39]. However, a disrupted alternative splicing process in multiple pre-mRNAs was further observed and altered proteins were related to clinical symptoms of DM1 such as insulin resistance, myotonia muscle wasting, cardiac abnormalities or cognitive deficits [40]. The splicing alterations in multiple related transcripts result mainly from an imbalance in the levels of RNA–binding splicing factors CUGBP1 (CUG binding protein 1, also named CUGBP and ETR3-like factor 1, CELF1) and MBNL1 (muscleblind-like 1). There are three types of MBNL in mammals: MBNL1, 2 and 3, with MBNL1 being the best characterised to date. In DM1, CUGBP1 is hyper-phosphorylated by protein kinase C (PKC) and its level increased, whereas MBNL1 and MBNL2 are bound abundantly to mutated transcripts and retained in the nucleus, co-localised with RNA foci and therefore are not accessible for a correct splicing process in other pre-mRNAs [41,42]. This “sequestration model” for MBNL is strongly supported by the results of the experiments in mouse models where the MBNL1 levels were increased after the transduction and the myotonia was reduced in HSA^LR^ poly(CUG) mice (human skeletal beta-actin long repeats = 250 “CUG” repeats) [43]. A similar effect manifested by the splicing aberration was observed *in vitro* in cells transfected with high concentrations of short synthetic oligoribonucleotides composed of “CUG” repeats [44]. The CUGBP1 protein interacts with different pre-mRNAs during muscle development and regulates the processing when phosphorylated by different kinases. The cyclin D3-cdk4/6 complex plays a key role in this process. In DM1 cells, the phosphorylation status of CUGBP1 and its interaction properties are modified due to a decreased level of cyclin D3 [45]. Jones *et al.* demonstrated that cyclin D3 is directed for proteasomal degradation by the GSK3β-mediated phosphorylation when GSK3β (glycogen synthase kinase 3β) is stabilised by an increased autophosphorylation in DM1 cells induced by the presence of “CUG” transcripts [46]. Additionally, there is an increased and sequestered RNA binding protein hnRNP H, which regulates the alternative splicing leading to aberrant splicing of insulin receptor (INSR) pre-mRNA in DM1 myoblasts [47].

Despite the length of the “CTG” repeat region, its transcription is not blocked and the expanded “CUG” sequences form hairpins or longer dsRNA structures within the transcripts. These RNA secondary structures were shown to be digested *in vitro* with ribonuclease Dicer to shorter (CUG)*_n_* sequences which may function as endogenous silencers (siRNA) of transcripts containing “CAG” repeats [48]. Moreover, the mutant transcripts interact with several transcription factors (SP1, STAT) disrupting cellular signalling and the transcription of target genes [49].

### 3.2. Huntington’s Disease Like 2

The expansion of “CUG” repeats (up to 41 repeats compared to normal cells harbouring 6–28 repeats) within the alternative exon 2a of Junctophilin-3 (*JPH-3*) mRNA leads to the progression of Huntington’s Disease Like 2 (HDL-2), an autosomal dominant disorder. The alternative splicing of exon 2a produces transcripts containing repetitions either within the coding region (translated into polyleucine or polyalanine tracts) or within the 3′ UTR. HDL-2, similarly to Huntington’s disease, manifests by motor defects, neurodegeneration and dementia [50,51]. Nevertheless, HDL-2 patients represent only a minor group of HD-like patients and are mostly Africans or with African remote ancestors [52]. The affected HDL-2 cells are characterised by the presence of RNA foci accumulating the *JPH-3* transcripts with bound MBNL1 resulting in an altered level of JPH-3 protein and in the neurodegeneration in the striatum [53]. Furthermore, a polyglutamine protein translated from antisense “(CAG)_n_” transcripts and accumulated into nuclear inclusions contributes to the pathogenesis via neuronal dysfunction in a mouse model [54]. However, this phenomenon may play a minor role in the progression of HDL-2 and the polyglutamine expanded proteins do not need to be detected in brain samples. Therefore, the sole reduced level of Junctophilin-3, perhaps in association with other cellular processes, might seriously contribute to HDL-2 progression as shown in *JPH-3* knockout mice [55].

## 4. Altered mRNA Secondary Structure

Mutations within the 3′ end might be responsible for the locally altered secondary structures of mRNAs and altered protein characteristics. GATA binding protein 4 (GATA4) is a transcription factor with zinc finger binding motifs that binds to the conserved “GATA” motifs within the promoter sequences of multiple genes [56]. It is essential for myocardial differentiation and function. Mutations found within the coding or non-coding regions lead to serious heart malformations, even death. Different mutations were detected within the 3′ UTR and predicted to alter the secondary structure of mRNA potentially affecting transport and localisation of mRNA and reduced binding of GATA4 to target DNA sequences. These reduced transactivations by GATA4 are assumed to directly participate in the congenital heart disease phenotype [56]. Congenital adrenal hyperplasia (CAH) is usually caused by the deficiency of the 21-hydroxylase enzyme encoded by the *CYP21A2* gene. However, in the non-classical form of CAH no mutations in *CYP21A2* were identified, suggesting an involvement of non-coding regulatory regions. Indeed, the *13 G→A substitutions within the 3′ UTR were identified in this subgroup of patients indicating that a substitution within the *CYP21A2* untranslated region is directly involved in the mild form of the disease probably, due to the altered secondary structure of pre-mRNA [57].

Modified regulatory characteristics of 3′ UTRs also result from polymorphisms within the specific *cis*–elements or outside them, both leading to the altered secondary structures of mRNAs that affect the accessibility of target binding sites to interacting *trans*-elements [58]. The A→C polymorphism within the miR-155 binding site within the 3′ UTR of *AGTR1* (Angiotensin II type 1 receptor) mRNA is responsible for a reduced binding of inhibitory miR-155 leading to an increased level of AGTR1 protein which is associated with hypertension. More than a weaker complementary sequence binding, the altered mRNA secondary structure and a lower accessibility to the miRNA target site may play role in this process [59]. The SNP rs9818870 (C→U polymorphism) in the *MRAS* 3′ UTR is associated with a reduced level of M-ras protein in patients with the coronary artery disease (CAD). A higher accessibility of miR-195 to its target site within the *MRAS* 3′ UTR and thus higher repression was shown in “U” allele due to a modified mRNA secondary structure even though the SNP is located within the sequence neighbouring the target site [59].

## 5. Cancer

Tumour development is generally accepted as a multistep process that involves genetic alterations resulting in the gain of oncogene activity and/or the loss of tumour suppressor gene function. Recent reports show that the alterations within 3′ UTRs such as mutations leading to the loss of miRNA complementary sites or changes in the length may significantly influence the expression of many genes [60–62]. In addition, many other regulatory sequences (*cis*-elements) within 3′ UTRs have been described to date and their alterations or incorrect interplay with specific binding proteins or RNAs (*trans*-elements) are also known to contribute to the malignant phenotype. Here we describe a few examples how variations in the pre-mRNA 3′ end processing step are linked with certain aspects of cancer.

Multiple pA sites are present in human pre-mRNA 3′ ends and an alternative processing represents another mechanism of the gene expression control [28,63]. The choice of a polyadenylation signal and an alternative cleavage and polyadenylation at more proximal or more distal pA site create mRNA isoforms of different 3′ UTR lengths that influence the expression levels mainly through the mRNA stability. Shorter 3′ UTR isoforms are generally more stable and produce higher protein levels compared to longer isoforms most likely due to the loss of negative regulatory *cis*-elements and the limited binding sites for miRNAs. Therefore, shorter 3′ UTR isoforms are more abundantly synthesised in proliferating and non-differentiated cells including cancer cells [31,64]. By analogy, transformed cells showing a high proliferation rate harbour short 3′ UTR isoforms synthesised in highly expressed oncogenes and this correlates with a worse prognosis of the disease [28,61,65].

Three different mutation mechanisms (A insertion at position 1176; 3 base deletion at position 942 and partial duplication of A-rich sequence at position 970) were detected to create a new polyadenylation site within the *CCND1* pre-mRNA. This alternative processing produces shorter and more stable cyclin D1 isoforms in mantle cell lymphoma leading to a higher proliferation rate and shorter survival of patients [66]. The most famous tumour suppressor, p53 protein, and its alterations are frequently discussed in relation to malignant transformation [67]. A SNP “AAUAAA”→“AAUACA” (rs78378222) was identified in the polyadenylation signal of *TP53* pre-mRNA disrupting the pre-mRNA processing and leading to decreased levels of p53 mRNA [68,69]. This transversion was found associated with different cancer types: basal cell carcinoma, prostate cancer, glioma, colorectal adenoma [68], oesophageal squamous cell carcinoma (ESCC) [69], diffuse large B-cell lymphoma (DLBCL) [70] or glioma [71]. Although this proposed mechanism potentially affecting p53 expression can contribute to the malignant phenotype, the role of rs78378222 variant and its prognostic value must be further elucidated. Recently, an alternative polyadenylation of *CDC6* (cell division cycle 6) pre-mRNA was shown to be induced by 17 β-oestradiol in oestrogen receptor positive breast cancers cells, revealing new aspects for the posttranscriptional control of gene expression [72].

The poly(A) polymerase (PAP), a member of the multiprotein complex arranging pre-mRNA processing, is mainly responsible for the addition of a poly (A) tail to the cleaved 3′ end. This step is crucial in the posttranscriptional regulation of the gene expression. As a higher rate of proteosynthesis is expected in proliferating cells, PAP was also shown to be more active in highly proliferating cells and furthermore more active in cancer cells compared to normal [73]. In this respect, the PAP activity level was shown to reflect the aggressiveness of breast carcinoma or leukaemia and represents an additional prognostic marker in breast carcinoma [74].

The adenylate uridylate (AU-rich) elements (AREs) are the most common regulatory elements within 3′ UTRs influencing the mRNA stability, the translation progress or the alternative RNA processing. AREs mostly destabilise mRNAs and repress the translation through a facilitation of the deadenylation process which results in the accelerated shortening of the poly(A) tail [75]. AREs may be disrupted by a mutation or reduced through the use of alternative PAS in many mRNAs whose elevated expression were related to proto-oncogene activation and a cancer progression. A proto-oncogene, *c-myc*, is constitutively expressed and the *c-myc* mRNA stabilised through a chromosomal translocation disrupting an ARE region in human plasma cell myeloma [76] and Burkitt lymphoma [77]. The enzyme cyclooxygenase 2 (COX-2) encoded by *PTGS2* (prostaglandin-endoperoxide synthase 2) gene is normally expressed at a low level or even absent because of an efficient regulation at the posttranscriptional level through a repressive ARE within 3′ UTR. Normally, ARE directs *PTGS2* mRNA to a rapid decay, and only under a pro-inflammatory or a growth-associated stimuli the COX-2 is rapidly elevated for an increased prostaglandin formation. An enhanced stability of a shorter *PTGS2* mRNA isoform resulting from the alternative cleavage of 3′ end and from the loss of repressive AREs was shown to be associated with chronic inflammatory diseases and colorectal cancer [78].

Certain types of cancer are known to be related to cellular transformation by oncogenic viruses. Human papilloma virus type 16 (HPV-16) infection belongs among the factors contributing to cervical cancer development and the viral transforming proteins E6 and E7 play key roles in carcinogenesis [79]. DNA of HPV-16 is often found integrated into the host genome in cancer cells [80,81]. Integrated viral DNAs were shown to express higher levels of E6/E7 mRNAs which were more stable than when expressed from extrachromosomal viral genomes. As the disruption of 3′ UTR of early viral region was detected in an integrated HPV-16, a disruption of a potential ARE sequence within this AU-rich region is suggested to participate significantly in this phenomenon [82].

## 6. Future Perspectives

Through this article we aimed to explain the importance of 3′ UTRs in the control of gene expression and show a tight relation of the 3′ UTR establishment and integrity to a functional proteosynthesis and a healthy cell. As the age of “RNA research” is still at the beginning, many questions about the roles of mRNA sequence/structure relationships and related RNA-binding proteins in the regulation processes remain to be elucidated.

Formerly, the exploration of mechanisms regulating gene expression was mainly oriented towards promoter regions and to related binding transcription factors and the 3′ untranslated regions, and their possible regulatory role at the posttranscriptional level were rather omitted. However, as many conserved sequences representing specific regulatory elements were discovered within the 3′ end, the 3′ UTRs were brought into focus during the last two decades. Different gene expression regulatory mechanisms related to 3′ UTRs were described to date, taking effect during cellular proliferation, differentiation, stress conditions and carcinogenesis as well. The disruption of the posttranscriptional events including effector protein complexes mostly represents a primary cause of 3′ UTR alteration, touching every step of the processing machinery. Secondly, mutations of regulatory sequences within 3′ UTRs and in proteins or ncRNAs (non-coding RNAs) binding to these sequence also occur and lead to a range of disorders in humans. The spectrum of 3′ UTR related disorders is wide and based on the results of recent studies may occur across all human diseases.
